# *In situ* beam reduction of Pu(IV) and Bk(IV) as a route to trivalent transuranic coordination complexes with hydroxypyridinone chelators

**DOI:** 10.1107/S1600577522000200

**Published:** 2022-02-25

**Authors:** Korey P. Carter, Jennifer N. Wacker, Kurt F. Smith, Gauthier J.-P. Deblonde, Liane M. Moreau, Julian A. Rees, Corwin H. Booth, Rebecca J. Abergel

**Affiliations:** a Chemical Sciences Division, Lawrence Berkeley National Laboratory, Berkeley, CA 94720, USA; b Department of Nuclear Engineering, University of California, Berkeley, CA 94720, USA

**Keywords:** plutonium, berkelium, coordination complexes, X-ray absorption spectroscopy, periodicity

## Abstract

The solution-state interactions of plutonium and berkelium with the octadentate chelator 3,4,3-LI(1,2-HOPO) (343-HOPO) were investigated and characterized by X-ray absorption spectroscopy, which revealed *in situ* reductive decomposition of the tetravalent species of both actinide metals to yield Pu(III) and Bk(III) coordination complexes. X-ray absorption near-edge structure (XANES) measurements were the first indication of *in situ* synchrotron redox chemistry as the Pu threshold and white-line position energies for Pu-343-HOPO were in good agreement with known diagnostic Pu(III) species, whereas Bk-343-HOPO results were found to mirror the XANES behavior of Bk(III)-DTPA. Extended X-ray absorption fine structure results revealed An—O_HOPO_ bond distances of 2.498 (5) and 2.415 (2) Å for Pu and Bk, respectively, which match well with bond distances obtained for trivalent actinides and 343-HOPO via density functional theory calculations. Pu(III)- and Bk(III)-343-HOPO data also provide initial insight into actinide periodicity as they can be compared with previous results with Am(III)-, Cm(III)-, Cf(III)-, and Es(III)-343-HOPO, which indicate there is likely an increase in 5*f* covalency and heterogeneity across the actinide series.

## Abstract 2

X-ray absorption spectroscopy was used to probe the interactions between an octadentate hy­droxy­pyridinone chelator and two transuranic elements in microgram quantities – plutonium and berkelium – within buffered solutions. Despite the precedence for chelation-driven stabilization of the tetravalent oxidation state of actinides with this ligand, *in situ* reductive decomposition yielded plutonium(III) and berkelium(III) coordination complexes.

## 1. Introduction

Over the past decade there has been a surge in interest in the coordination chemistry of trivalent and tetravalent transuranic elements due to the distinctive chemical properties of the actinides, increased availability of materials, and improvements in instrumentation development and data processing (Carter, Pallares *et al.*, 2020 ▸), particularly for X-ray absorption spectroscopy (XAS) and diffraction techniques (Galbis *et al.*, 2010 ▸; Cary *et al.*, 2015 ▸; Ferrier *et al.*, 2017 ▸; Kelley *et al.*, 2018 ▸; Müller *et al.*, 2021 ▸; Jones *et al.*, 2021 ▸). Recent highlights of these efforts include the first crystal structure of Bk(III) (Silver *et al.*, 2016 ▸), stabilization of Bk(IV) in aqueous solution (Deblonde *et al.*, 2017 ▸), measurement of the first bond distance with Es(III) (Carter, Shield *et al.*, 2021 ▸), and a significant improvement in our understanding of how covalency and heterogeneity affect 5*f*-orbital bonding (Allred *et al.*, 2015 ▸; Cross *et al.*, 2017 ▸; Kelley *et al.*, 2017 ▸; Su *et al.*, 2018 ▸; Stein *et al.*, 2019 ▸; Wilson *et al.*, 2020 ▸; Bessen *et al.*, 2021 ▸). Even with these advances, transuranic coordination chemistry remains largely underdeveloped, and there is a need to further delineate trends in structure, bonding, and periodicity, as well as evaluate metal–ion redox stability under a range of conditions.

Multidentate hy­droxy­pyridinone (HOPO) and catecholamide (CAM) ligands are a class of ligands known to chelate both actinide and lanthanide cations exceptionally well. In addition, they have also been shown to be effective *in vivo* decorporation agents of trivalent and tetravalent actinides (Gorden *et al.*, 2003 ▸; Kullgren *et al.*, 2013 ▸; Captain *et al.*, 2016 ▸; Abergel, 2017 ▸; Ricano *et al.*, 2019 ▸; Arnedo-Sanchez *et al.*, 2021 ▸; Pallares *et al.*, 2021 ▸). Exemplary in these efforts is the octadentate chelator 3,4,3-LI(1,2-HOPO), denoted 343-HOPO hereafter, which has been studied with a range of trivalent and tetravalent *p*-, *d*- and *f*-block metals (Abergel *et al.*, 2009 ▸; Sturzbecher-Hoehne *et al.*, 2011 ▸; Deblonde *et al.*, 2013 ▸, 2017 ▸; Deblonde, Lohrey *et al.*, 2018 ▸; Carter, Deblonde *et al.*, 2020 ▸; Carter, Shield *et al.*, 2021 ▸). In both coordination chemistry and *in vivo* systems, there are significant indications that 343-HOPO chelation-driven redox chemistry is possible (Xu *et al.*, 2000 ▸; Deblonde *et al.*, 2013 ▸, 2017 ▸, 2019 ▸; Carter *et al.*, 2018 ▸; Carter, Smith *et al.*, 2020 ▸, 2021 ▸), wherein oxidation or reduction of the metal ions are induced depending on redox couple accessibility and complexation thermodynamics. Stability of the resulting complexes in solution has not been fully developed, however, as shown by the recent example of X-ray beam-induced reduction of a Ce(IV)-343-HOPO complex during XAS measurements (Bailey *et al.*, 2021 ▸).

Herein, we detail two additional examples of reductive decomposition of tetravalent plutonium and berkelium complexes during *L*_III_-edge XAS experiments, which are likely the result of radiolysis within the sulfonic acid buffer matrices induced from the intense high-energy X-ray beam. Although we recently used solution-state XAS methodologies to characterize both trivalent and tetravalent aqueous actinide systems (Deblonde, Kelley *et al.*, 2018 ▸; Kelley *et al.*, 2018 ▸; Carter, Smith *et al.*, 2020 ▸, 2021 ▸; Carter, Shield *et al.*, 2021 ▸), we have not done so with redox-active ligands, such as 343-HOPO, and metals with an accessible +IV/+III redox couple. Plutonium and berkelium are two transuranic actinides that meet these criteria, with standard +IV/+III redox couples of 0.97 and 1.60 V (in 1 *M* HClO_4_ versus NHE) for Pu and Bk, respectively (Boukhalfa *et al.*, 2007 ▸; Antonio *et al.*, 2002 ▸). Detailed solution-state electronic and structural information for both Pu(III) and Bk(III) complexes with 343-HOPO were obtained via XANES and extended X-ray absorption fine structure (EXAFS) spectroscopies upon reductive decomposition of tetravalent Pu and Bk complexes. These results are particularly notable as Pu(III) and Bk(III) solution-state complexes are relatively rare, with XAS being a key characterization tool that has developed our understanding of the chemical behavior of both species. Pu(III) systems are more prevalent compared with Bk(III), in which XAS studies have explored the interactions of Pu(III) with simple aquo and inorganic monodentate and bidentate ligands (Allen *et al.*, 1997 ▸, 2000 ▸; Ankudinov *et al.*, 1998 ▸; Conradson *et al.*, 2004 ▸; Popa *et al.*, 2015 ▸; Vitova *et al.*, 2018 ▸; Brendebach *et al.*, 2009 ▸), organic chelators (Audras *et al.*, 2017 ▸; Arab-Chapelet *et al.*, 2016 ▸) and in environmentally relevant systems (Dardenne *et al.*, 2009 ▸; Kirsch *et al.*, 2011 ▸; Schmeide *et al.*, 2006 ▸; Marquardt *et al.*, 2004 ▸). In contrast, only two examples of XAS studies to characterize Bk are known in either the Bk(III) or Bk(IV) oxidation state (Antonio *et al.*, 2002 ▸; Deblonde, Kelley *et al.*, 2018 ▸).

## 2. Experimental methods

Caution! ^249^Bk (*t*_1/2_ = 330 days, 61 T Bq g^−1^) and ^249^Cf (*t*_1/2_ = 351 years, 150 GBq g^−1^) are highly radioactive β- and α-emitting radionuclides, respectively, and decay to α- and γ-emitting isotopes, whereas ^242^Pu (*t*_1_ = 3.75 × 10^5^ years, 0.15 GBq g^−1^) is an α-emitting radionuclide. These isotopes, as well as their decay daughters, present significant health risks and were manipulated only in facilities specifically designed for the safe handling of long-lived radioactive materials. All measurements were taken either in controlled facilities and/or using multiple containment procedures.

### 2.1. Materials

The ligand 343-HOPO was prepared and characterized as previously described (Abergel *et al.*, 2010 ▸) and ligand stock solutions were assembled by direct dissolution of weighted portions into water or di­methyl­formamide (DMF). ^249^BkCl_3_ and ^242^PuO_2_ were received from Oak Ridge National Laboratory, and a stock solution of ^242^Pu(IV) was prepared as described previously (Gorden *et al.*, 2007 ▸). All other chemicals used in this study were obtained from commercial suppliers and were used as received.

### 2.2. Preparation of XAS samples

#### 2.2.1. Pu-343-HOPO

The Pu-343-HOPO XAS sample was assembled from aliquots of the metal and ligand stock solutions with a metal:ligand ratio of 1:2 and the final Pu concentration was 1 m*M*. Additionally, 5 µl of DMF was added to ensure solubility of the metal–chelate complex. The pH of the sample was buffered to 7–8 using 50 m*M* HEPES and approximately 65 µl was loaded into indium- and ep­oxy-sealed, triply contained, aluminium holders with Kapton windows (developed in-house) within ten days of synchrotron measurement (Pugmire *et al.*, 2019 ▸).

#### 2.2.2. Bk-343-HOPO and Bk-DTPA

Bk-343-HOPO and Bk-DTPA XAS samples were previously prepared as part of samples containing both ^249^Bk and ^249^Cf (Deblonde, Kelley *et al.*, 2018 ▸; Kelley *et al.*, 2018 ▸), and details are included here for completeness. For both 343-HOPO and DTPA, samples containing both ^249^Bk(III) and ^249^Cf(III) (ratio Cf/Bk = 1.9) were prepared using a ^249^BkCl_3_ stock solution in 0.1 *M* HCl that had decayed for 510 days to allow for in-growth of the ^249^Cf daughter. The Bk/Cf samples were prepared using a metal:ligand ratio of 1:1.3 to ensure complete metal–ion complexation and assembled from aliquots of the metal stock solution with either a 343-HOPO stock solution in water or a DTPA stock solution at pH 4. The metal–ligand complexes were diluted with CAPS buffer (Sigma–Aldrich, BioUltra, >99%) to volumes of ∼65 µl to yield final concentrations of 0.11 m*M* for ^249^Bk and 0.20 m*M* for ^249^Cf. The pH values of the samples were buffered to 7–8 and each solution was loaded into separate, triply contained, aluminium holders (analogous to Pu) within ten days of synchrotron measurement.

### 2.3. XAS data collection and processing

XANES and EXAFS spectroscopy data were collected at the Pu and Bk *L*_III_-edges on beamline 11-2 at the Stanford Synchrotron Radiation Lightsource (SSRL) using an Si(220) (ϕ = 0°) double-crystal monochromator detuned by 50%. Samples were held in an LHe-cooled cryostat at 50 K throughout analysis and all XAS measurements were collected in fluorescence mode using a 100-element Canberra Ge detector and corrected for dead-time. Pu and Bk XANES spectra were calibrated to Zr (*K*-edge, 17995.88 eV) and Mo (*K*-edge, 20000.36 eV) standards, respectively (Kraft *et al.*, 1996 ▸). Processing of the data is described in the supporting information, including an important discussion on multiple scattering.

## 3. Results

### 3.1. Synthesis of An-343-HOPO complexes

**Figure 1:**
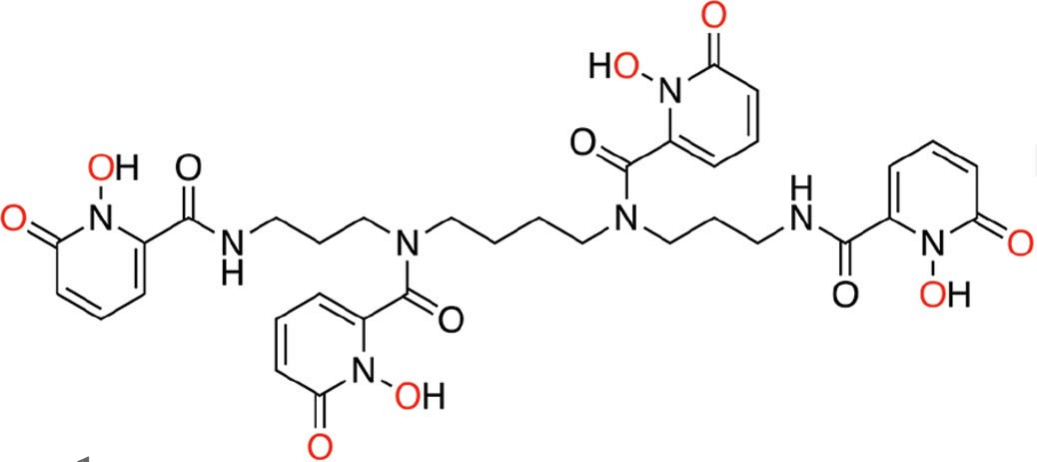
Molecular structure of 343-HOPO. Oxygen atoms expected to bind to the actinide ions are highlighted in red.

Acidic stock solutions of ^242^Pu(IV) and ^249^Bk(III) were combined with a slight excess of the 1-hy­droxy-2-pyridinone ligand (343-HOPO; Fig. 1 ▸) and buffered to a pH value of 7–8 to ensure metal binding. No efforts were made to exclude air or moisture. Final concentrations – based on the metal – of Pu-343-HOPO and Bk-343-HOPO samples were 1 m*M* (19.4 µg) and 0.11 m*M* (1.7 µg), respectively. Both Pu and Bk were expected to be fully coordinated to the deprotonated ligand, bound through the oxygen atoms of carbonyl and hydroxyl groups of the hy­droxy­pyridinone moiety to form octadentate coordination complexes (Sturzbecher-Hoehne *et al.*, 2015 ▸). Additionally, we anticipated both Pu and Bk ions to be in the +IV oxidation state, akin to previous studies in our laboratory that have demonstrated the chelation-driven stabilization of Ln(IV) and An(IV) metal-ions by 343-HOPO in solution (Deblonde *et al.*, 2013 ▸, 2017 ▸, 2019 ▸; Sturzbecher-Hoehne *et al.*, 2015 ▸; Carter, Smith *et al.*, 2020 ▸, 2021 ▸). To analyze Pu and Bk oxidation states and complexation behaviors with 343-HOPO, we used XAS, which is uniquely positioned to assess the transuranic actinide elements, particularly those where limited mass quantities are available, such as Bk.

### 3.2. XANES measurements of Pu-343-HOPO and Bk-343-HOPO

**Figure 2:**
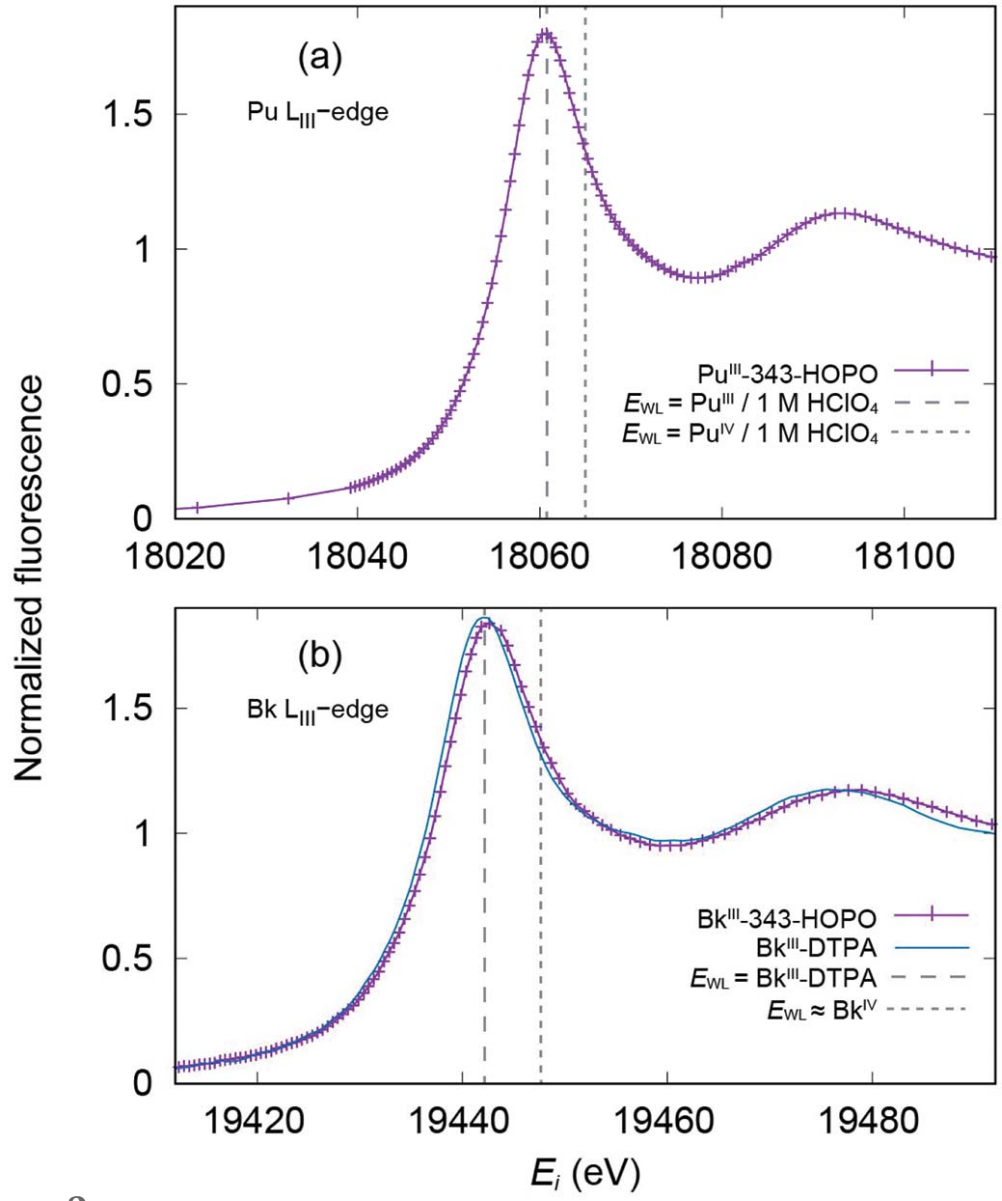
*L*_III_-edge XANES spectra collected on frozen solutions of (*a*) Pu(III)-343-HOPO and (*b*) Bk(III)-343-HOPO (purple traces). The vertical dashed lines in (*a*) highlight the white-line position (*E*_WL_) for oxidation-state references Pu(III) and Pu(IV) in 1 *M* HClO_4_ solutions (Conradson *et al.*, 2004 ▸), which have been calibrated to the Zr *K*-edge (Kraft *et al.*, 1996 ▸). The XANES data for Bk(III)-DTPA (blue trace) is plotted in (*b*) for comparison, along with the Bk(III)-DTPA *E*_WL_ position (vertical dashed line). In addition, an estimated *E*_WL_ position of a hypothetical Bk(IV)-DTPA complex (vertical dashed line) is shown as a Bk(IV) oxidation state reference. The estimate of the Bk(IV)-DTPA *E*_WL_ was calculated as a +5.5 eV shift from the Bk(III)-DTPA white line as was observed in the electrochemical oxidation of Bk(III) to Bk(IV) in 1 *M* HClO_4_ acidic solution (Antonio *et al.*, 2002 ▸).

XANES measurements were collected on the prepared actinide solutions to determine the oxidation states of the Pu and Bk metal cations in the presence of 343-HOPO; Fig. 2 ▸ shows the calibrated An *L*_III_-edge XANES data for both samples. Despite expectations of Pu and Bk to be tetravalent in the presence of 343-HOPO, the XANES data instead strongly suggest both metals are in the +III oxidation state, and as such are denoted hereafter Pu(III)-343-HOPO and Bk(III)-343-HOPO. The threshold energy *E*_0_, as measured by the energy of the peak in the first derivative, is 18057.4 ± 0.1 eV for the buffered solution of ^242^Pu(IV) and 343-HOPO, while the white-line position *E*_WL_, or rather the position of the peak in the spectrum, is found at 18060.7 ± 0.1 eV by fitting to the experimental data. The analogous values obtained for the buffered solution of ^249^Bk(III) and 343-HOPO are located at *E*_0_ = 19438.1 ± 0.1 eV and *E*_WL_ = 19442.9 ± 0.1 eV, respectively.

The XANES metrical parameters are comparable with previous reports of these actinides in the trivalent oxidation state. Conradson *et al.* (2004 ▸) demonstrated that plutonium *E*_0_ values can vary by up to 1 eV within a single oxidation state of Pu, subject to effects from the matrix environment, local disorder, and ligand coordination. A selection of standards from Conradson *et al.* (2004 ▸) have been calibrated to the Zr *K*-edge reported by Kraft *et al.* (1996 ▸) and compared with Pu(III)-343-HOPO. In particular, Pu(III) in perchlorate (1 *M*) solution, solid-state Pu(IV)O_2_, and Pu(IV) in perchlorate (1 *M*) solution have *E*_0_ values of 18056.5, 18058.8, and 18059.7 eV, and white-line positions at 18060.8, 18065.1, and 18065.2 eV, respectively. By utilizing these literature reports as a basis for oxidation-state assignment, the Pu(III)-343-HOPO data – with *E*_0_ equal to 18057.4 ± 0.1 eV and an *E*_WL_ value at 18060.7 ± 0.1 eV – are consistent with Pu(III) rather than Pu(IV).

By comparison with Pu and other actinides, Bk XANES data are very rare in the literature. In fact, there has only been one other report by Antonio *et al.* (2002 ▸) where XAS was used to study Bk speciation. Unfortunately, this pioneering work was performed within an electrochemical cell that had poor instrumental resolution, and therefore it is difficult to compare with our results. While Antonio *et al.* (2002 ▸) could not obtain an absolute measurement of *E*_0_ and *E*_WL_, they did observe a relative separation of +5.5 eV when Bk was oxidized from +III to +IV in acidic aqueous solution. We can compare our measurements with Bk(III) in the presence of the DTPA ligand, which does not have the same oxidative control as 343-HOPO. Therefore, Bk is expected to remain in its most stable oxidation state (+III) upon complexation to DTPA. These data were originally used only for EXAFS analysis by Deblonde, Kelley *et al.* (2018 ▸) and are shown here for the first time in Fig. 2 ▸(*b*). Bk(III)-DTPA has an *E*_0_ = 19438.2 ± 0.1 eV and an *E*_WL_ = 19442.2 ± 0.1 eV, and agreement between Bk(III)-343-HOPO and Bk(III)-DTPA white-line positions strongly support the assignment of the +III oxidation state in the Bk(III)-343-HOPO sample. Taken together with Pu(III)-343-HOPO data, these results indicate reductive decomposition of the tetravalent Pu and Bk complexes during *L*_III_-edge XAS experiments despite the presence of, and chelation by, 343-HOPO.

### 3.3. Examination of actinide coordination behavior via EXAFS

**Figure 3:**
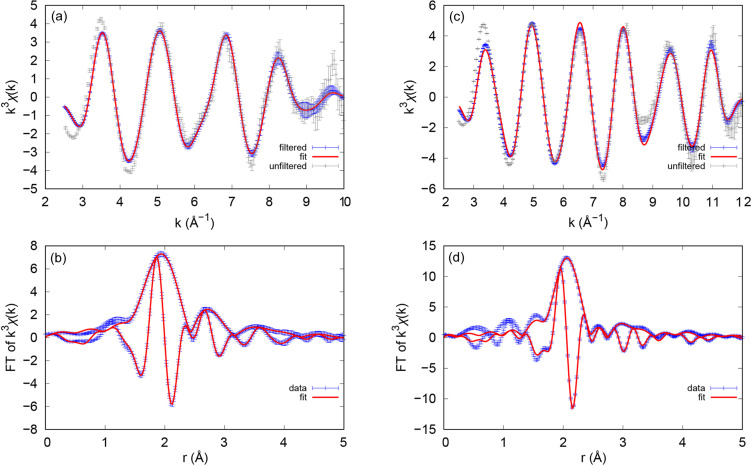
EXAFS data and fit (top) and Fourier transform of the *k*-space data and fit (bottom) for Bk(III)-343-HOPO and Pu(III)-343-HOPO. (*a*, *b*) Free fit, except fixed multiple scattering peak amplitudes, for Bk(III)-343-HOPO. (*c*, *d*) Pu(III)-343-HOPO fit with multiple scattering paths fixed. Error bars for the raw, unfiltered data are estimated as the standard deviation of the mean between individual traces. Note that comparison between fit and data in *k*-space should be to the filtered data, which has been back-transformed over the fit range.

EXAFS measurements were collected on the prepared actinide solutions to not only supplement Pu and Bk oxidation-state assignments but also to improve our understanding of the coordination behavior and periodicity of these transuranic elements through the lens of 343-HOPO chelation. As the Bk data were simpler to handle with regards to multiple scattering (MS; see processing details in the supporting information), we first detail these results, obtained from the wavevector *k*, of 2.5 to a maximum of 10.0 Å^−1^. Figs. 3 ▸(*a*) and 3 ▸(*b*) show the *k* ^3^-weighted Bk *L*_III_-edge EXAFS spectra and corresponding Fourier transforms with all fitting parameters included in Table S1 of the supporting information. The fit for Bk(III)-343-HOPO converges easily with or without constraining the amplitudes to the coordination numbers; metrical parameters of note include a coordination number (*N*) equal to 8.1 (3), consistent with complete 343-HOPO binding to yield the anionic complex [Bk(III)(343-HOPO)]^−^. Found by fixing the amplitude reduction factor to *S*_0_^2^ = 1.0 [akin to Bk(III)-DTPA (Kelley *et al.*, 2018 ▸)], the *N* value is obtained from a preliminary fit assuming nominal values for each *N*, and is consistent with other An(III)-343-HOPO species (Kelley *et al.*, 2018 ▸). Additionally, the average Bk–O_HOPO_ bond length was found to be 2.415 (2) Å with a Debye–Waller factor of σ^2^ = 0.0094 (4). DFT calculations from Kelley *et al.* (2018 ▸) were used to understand the interactions between Bk and 343-HOPO in water, estimating a Bk(III)—O_HOPO_ distance of 2.43 Å and Bk(IV)—O_HOPO_ of 2.37 Å. As the calculated Bk(III)—O_HOPO_ bond distances are consistent with those observed in Bk(III)—343-HOPO, the EXAFS fit therefore supports the identification of a Bk(III) species. This fit also demonstrates that the degree of multiple scattering observed in experimental data is similar to that in DFT structure calculations (Kelley *et al.*, 2018 ▸), yet it should be noted that excluding MS does not change the results from the other shells significantly.

Despite challenges related to multiple scattering (outlined in the supporting information), high-quality Pu(III)-343-HOPO EXAFS data were obtained from the wavevector *k* of 2.5 to a maximum of 12.0 Å^−1^. Figs. 3 ▸(*c*) and 3 ▸(*d*) show the *k* ^3^-weighted Pu *L*_III_-edge EXAFS spectra and corresponding Fourier transforms with all fitting parameters included in Table S3. This fit also constrains the coordination numbers to avoid correlations between the MS path and the Pu–C/N shell. Because of the role of MS in the Pu(III)-343-HOPO data, the degree of 343-HOPO coordination is not as conclusive as with Bk(III)-343-HOPO, although the data are consistent with full coordination and a Pu(III)—O_HOPO_ bond distance of 2.498 (5) that aligns with previous reports of Pu(III) solution studies (Conradson *et al.*, 2004 ▸). DFT calculations from Kelley *et al.* (2018 ▸) were also used to understand the interactions between Pu and 343-HOPO in water, estimating a Pu(III)—O_HOPO_ distance at 2.46 Å that is lengthened compared with Pu(IV) (2.38 Å), which is consistent with the Pu—O_HOPO_ bond distance observed in Pu(III)-343-HOPO. Thus, the EXAFS fit supports the identification of a Pu(III) species.

## 4. Discussion

XANES and EXAFS measurements were leveraged to understand the complexation of two transuranic elements, Pu and Bk, with the chelator 343-HOPO in buffered solutions. Despite the precedent of chelation-driven redox chemistry as a pathway to stabilize the tetravalent oxidation state of these elements (Sturzbecher-Hoehne *et al.*, 2015 ▸; Deblonde *et al.*, 2017 ▸), reductive decomposition of Pu(IV)-343-HOPO and Bk(IV)-343-HOPO was observed in XAS measurements to yield Pu(III)-343-HOPO and Bk(III)-343-HOPO. These studies provide a rare example of trivalent Pu(III) and Bk(III), wherein the oxidative controls of 343-HOPO were outmatched by external factors, namely high-flux synchrotron radiation at an energy of ∼18000–19000 eV. The XANES spectrum collected for Pu(III)-343-HOPO illustrates *E*_0_ and white-line energies that align with those expected for Pu(III). Although matrix effects can influence these energies, when combining the XANES results with the EXAFS model showing a Pu—O_HOPO_ distance of ∼2.5 Å, the data strongly suggest Pu(III) is the dominant oxidation state in Pu(III)-343-HOPO. Oxidation-state determination of Bk is inherently more challenging, simply because there are only two other examples of Bk XAS data (Antonio *et al.*, 2002 ▸; Deblonde, Kelley *et al.*, 2018 ▸). As such, we supported the assignment of Bk(III)-343-HOPO by direct comparison with a known Bk(III) compound, Bk(III)-DTPA.

In both cases, 343-HOPO was found to coordinate Pu(III) and Bk(III) to yield octadentate complexes with first coordination sphere An(III)—O_HOPO_ distances of 2.498 (5) and 2.415 (2) Å, respectively. We note that Pu-343-HOPO EXAFS has been studied once previously, but with samples prepared under very different conditions from those used in this work and with less emphasis on the XANES features, and, as such, precludes meaningful comparisons (Aupiais *et al.*, 2017 ▸). We hypothesize the Pu and Bk reduction likely resulted from radiolysis provided by the high-energy X-ray beam. In addition, sulfonic acid buffers – the matrices in which these measurements were collected – are known to enhance or facilitate redox activity (Cuculić *et al.*, 1998 ▸), yet it should be noted that reductive decomposition of Pu and Bk was also observed in other buffers (*i.e.* TRIS buffer). Similar reductive decomposition was also recently observed in lanthanide systems with Ce(IV)-343-HOPO reduction to Ce(III)-343-HOPO (Bailey *et al.*, 2021 ▸). Photoreduction due to the X-ray beam has also been reported for transition metals (van Schooneveld & DeBeer, 2015 ▸), such as iron +III/+II, ruthenium +IV/+III and manganese +III/+II transformations (Gonçalves Ferreira *et al.*, 2013 ▸; Mo *et al.*, 2000 ▸; Risch *et al.*, 2017 ▸). Taking into account these examples of reductive beam effects on transition metal, lanthanide and now transuranic systems, caution is advised when conducting oxidation-state studies at the *L*_III_-edge for Pu and Bk samples. Recently, efforts to capture and characterize *in situ* Pu(IV) and Bk(IV) reductions were attempted at SSRL wherein beam energies were kept stationary at the relative *L*_III_-edge energies of Pu(IV) and Bk(IV) (∼18067 and ∼19444 eV, respectively) and the change in energy as a function of time was monitored. It was hypothesized that a shift to lower energy would be observed, consistent with reduction of the actinide metal ion from the +IV to the +III oxidation state and similar to the reduction of Ce(IV)-343-HOPO to Ce(III)-343-HOPO identified in previous studies (Bailey *et al.*, 2021 ▸). However, no substantial change in the *L*_III_-edge energy intensities was detected as a function of time (*t* = 10 min; data points collected every 1 s). Subsequent XANES scans indicated absorption edges consistent with +III species, and therefore we postulate that the reduction occurred faster than the timescale of the initial data point.

**Figure 4:**
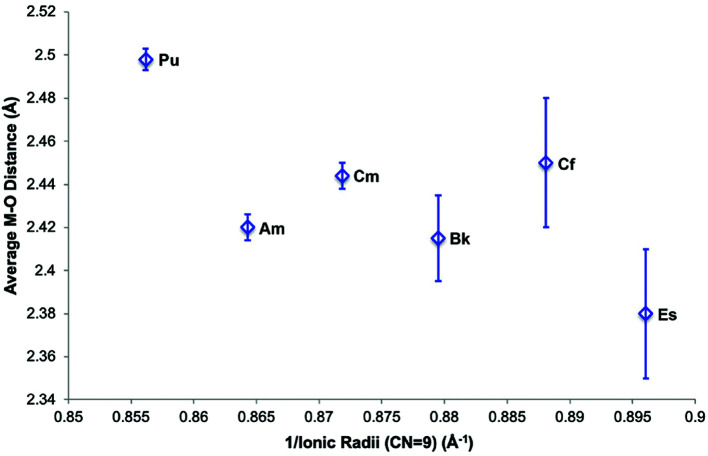
Comparison of An—O_HOPO_ bond distances relative to the ionic radii of trivalent, transuranic actinides Pu–Es. Ionic radii were taken from Lundberg & Persson (2016 ▸), while Am, Bk, Cf, and Es data have been published previously (Kelley *et al.*, 2018 ▸; Carter, Shield *et al.*, 2021 ▸). Error bars are based on a profiling method from the fits, together with an estimate of the probable systematic error in the Cf and Es data (Carter, Shield *et al.*, 2021 ▸).

Fortuitously, *in situ* production of Pu(III) and Bk(III) allowed for detailed solution-state electronic and structural information to be obtained on transuranic species. Moreover, Pu(III)-343-HOPO and Bk(III)-343-HOPO fit into an extended series of An(III)-343-HOPO complexes that have been reported by our group. Additions of Pu(III) and Bk(III) to this series result in an exceptional and uncommon dataset: coordination complexes of the trivalent actinides spanning Pu to Es (Kelley *et al.*, 2018 ▸; Carter, Shield *et al.*, 2021 ▸). Put into a larger picture, these data inform of periodicity across the 5*f* series, exhibiting a general shortening of the An—O_HOPO_ bonding interaction that is consistent with the actinide contraction. More broadly, additions of experimental data points to the An(III)-343-HOPO series can provide insight into an increase in covalency and heterogeneity as one moves farther along in the actinide series (Fig. 4 ▸), and is the subject of ongoing investigations.

## 5. Conclusions and outlook

In summary, *in situ* reductive decomposition of Pu(IV) and Bk(IV) was observed during XAS measurements, which yielded Pu(III) and Bk(III) coordination complexes with the octadentate chelator 343-HOPO. XANES and EXAFS spectroscopies on Pu- and Bk-343-HOPO samples confirmed metal ion reduction and chelation, providing rare insight into the coordination chemistry behavior of both trivalent species. Limited XAS studies with Pu(III) have focused on aqueous inorganic, organic, or environmentally relevant ligand systems (Allen *et al.*, 1997 ▸, 2000 ▸; Ankudinov *et al.*, 1998 ▸; Arab-Chapelet *et al.*, 2016 ▸; Popa *et al.*, 2015 ▸; Vitova *et al.*, 2018 ▸; Brendebach *et al.*, 2009 ▸; Kirsch *et al.*, 2011 ▸; Schmeide *et al.*, 2006 ▸; Marquardt *et al.*, 2004 ▸) and only two XAS experiments have been conducted on Bk previously (Antonio *et al.*, 2002 ▸; Deblonde, Kelley *et al.*, 2018 ▸). As such, results included herein provide necessary metrical parameters to better assess future Pu(III) and Bk(III) coordination complexes in condensed aqueous phases. Pu(III)-343-HOPO and Bk(III)-343-HOPO bond distances were found to agree with previous DFT calculations for +III complexes and were compared with the results of Am(III), Cm(III), Cf(III), and Es(III) in the presence of 343-HOPO. Consequently, insights into actinide periodicity were demonstrated that can inform the extent to which 5*f* covalency and heterogeneity increase across the actinide series. Overall, these results highlight the ability to use XAS to assess microgram quantities of actinide complexes, even in less-common oxidation states, which has greatly expanded our understanding of the 5*f* block, and which we will continue to build on in future studies focused on actinide chelation with bio-inspired ligands.

## 6. Related literature

The following references, not cited in the main body of the paper, have been cited in the supporting information: Ankudinov *et al.* (1998 ▸); Booth & Bridges (2021 ▸); Booth & Hu (2009 ▸); Li *et al.* (1995 ▸).

## Supplementary Material

Supporting figures and tables. DOI: 10.1107/S1600577522000200/ok5066sup1.pdf
