# Supplementary material for: In situ beam reduction of Pu(IV) and Bk(IV) as a route to trivalent transuranic coordination complexes with hydroxypyridinone chelators
Source: J Synchrotron Radiat. 2022 Feb 25;29(Pt 2):315–22. doi: 10.1107/S1600577522000200 (PMC8900832; doi:10.1107/S1600577522000200)
Supplement: Supplementary file 1 [file s-29-00315-sup1.pdf]

JOURNAL OF SYNCHROTRON RADIATION

Volume 29 (2022)

Supporting information for article:

In situ beam reduction of Pu(IV) and Bk(IV) as a route to trivalent transuranic coordination complexes with hydroxypyridinone chelators

Korey P. Carter, Jennifer N. Wacker, Kurt F. Smith, Gauthier J.-P. Deblonde, Liane M. Moreau, Julian A. Rees, Corwin H. Booth and Rebecca J. Abergel

S1. X-ray Absorption Spectroscopy Data Processing Details

Extended X-ray absorption fine structure (EXAFS) data reduction and analysis were conducted using the RSXAP software suite (Booth & Bridges, 2021, Li *et al.*, 1995, Booth & Hu, 2009) in conjunction with backscattering line shapes and phases calculated using FEFF8.5L (Ankudinov *et al.*, 1998). Data were transformed between a wave vector (k) of 2.5 and 12.0 Å^{-1} (for Pu) and 2.5 and 10.0 Å^{-1} (for Bk) using a 0.3 Å^{-1} wide Gaussian window and all fitting was conducted in r-space. Error analysis was performed using a profiling method (Booth & Hu, 2009) and for fits of Pu- and Bk-343-HOPO the total number of fitting parameters was less than two-thirds the total number of independent points.

Multiple scattering (MS), especially for Pu-343-HOPO, was found to affect EXAFS fit models and required treatment beyond normal standards, thus a short discussion is provided to explain how MS was addressed for each sample. For Bk-343-HOPO, MS from the FEFF model was treated as a single scattering standard, which combined all effective MS path lengths less than 4 Å. The amplitude was then fixed by S_0^2 and an overall Debye-Waller factor and path shift were allowed to float in the fit. Similar treatment was not satisfactory for Pu, a likely result of the calculated structure of Pu-343-HOPO containing various multiple scattering paths with similar effective lengths, including 16 three-leg and four-leg scattering paths all between 3.5 and 3.6 Å and 14 such paths between 3.7 and 3.8 Å, after which a near continuum of paths exist well beyond the limits of our useful data range. The MS paths shorter than 3.8 Å pertain to photoelectrons originating from the absorbing actinide and then scattering off the nearest O, N, and C neighbors (e.g., Pu-O-C-Pu, a 3-leg path). As a result, the MS calculation of the Pu-343-HOPO is more sensitive to the details of the actual structure than that of the Bk-343-HOPO, and therefore the MS FEFF calculations could be miscalculated. We observe a manifestation thereof when Pu-343-HOPO data is fit and the number of multiple scattering paths are fixed in a similar way to the Bk-343-HOPO model (Figure S1, Table S2), wherein there is unaccounted amplitude near 3.3 Å in the Fourier transform. To address this shortcoming, we had to first identify the extent of the FEFF calculation error due to MS, which was done by allowing the MS amplitude to vary while constraining coordination numbers of other paths. This yielded

an estimate of an approximately 1.7x greater MS contribution than calculated by FEFF, which was accounted for in the final fit of Pu-343-HOPO using a single Pu-O-N-Pu path reported below.

S2. Additional Figures and Tables

Table S1 **EXAFS fit results from Bk^{III}–343–HOPO**. Fit range is between 1.5 and 5.0 Å. The k^3-weighted data are transformed between 2.5-10.0 $Å^{-1}$ and are Gaussian narrowed by 0.3 $Å^{-1}$. The data have 18.7 independent data points. The fit has 4.7 degrees of freedom. Also shown are DFT results for the ground state Bk^{IV}-343-HOPO from Kelley *et al*. (Kelley *et al.*, 2018). Where the Debye-Waller factor σ^2 is reported for the EXAFS data, which includes both static and thermal contributions to the bond length distribution width, "var" represents the variance in the calculated bond length distribution for the given shell, and includes no thermal contribution. The MS peak is modeled from all the MS paths shorter than 4 Å derived by FEFF for the DFT structure. Note that values without reported errors are fixed in the fit. A single amplitude reduction factor, S_0^2, was used in determining the coordination numbers (N).

	N (Nominal)	DFT		EXAFS		
		var ($Å^2$)	R (Å)	N	σ^2 ($Å^2$)	R (Å)
Bk–O	8	0.0010	2.358	8.1(3)	0.0094(4)	2.415(2)
Bk–C/N	8	0.0011	3.224	8(1)	0.010(2)	3.251(4)
Bk–C	10	0.0135	4.609	9(6)	0.007(5)	4.73(2)
MS	36	0.042	3.708	36	0.002(1)	3.827(9)
					ΔE_0 = -19.3(3) eV<br>S_0^2 = 1.0<br>R(%) = 2.2	

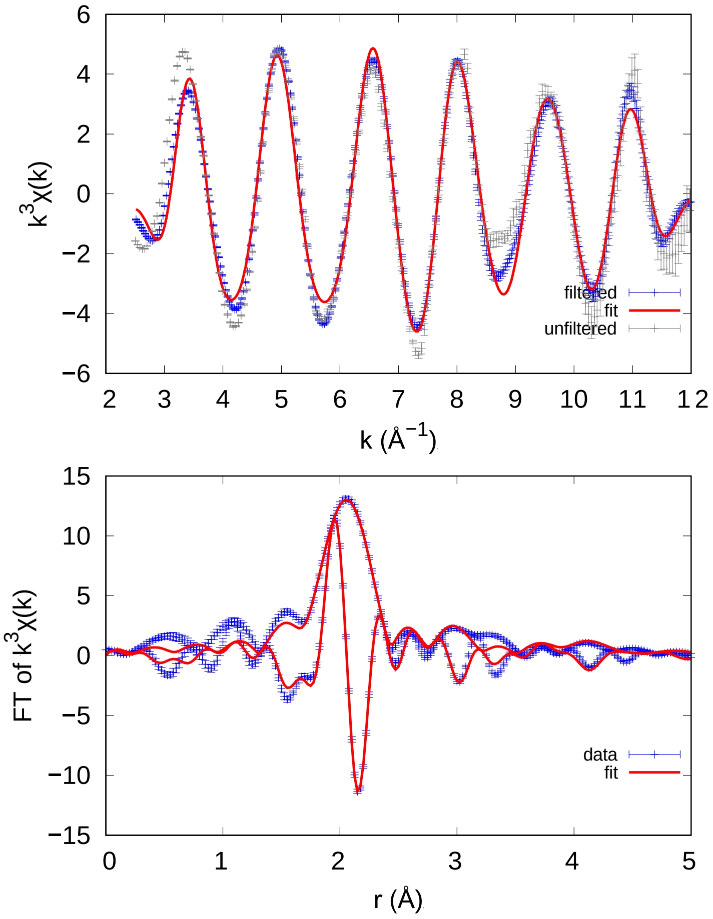

Figure S1 EXAFS data and fit (top) and Fourier transform of the *k*-space data and fit (bottom) for Pu^{III}–343–HOPO, holding the number of MS paths fixed to the nominal value from DFT calculations. Fits correspond to the metrical parameters presented in Table S2. Note poor fit quality near 3.3 Å in the transform.

Table S2 Fit results from Pu^{III}–343–HOPO modelled with the coordination number, N, for the multiple scattering paths fixed to the nominal value from DFT calculations (see Table S3). The Debye-Waller factor is referred to here as σ^2. The fit range is between 1.5 and 5.0 Å. The k^3-weighted data are transformed between 2.5-12.0 $Å^{-1}$ and are Gaussian narrowed by 0.3 $Å^{-1}$. The data have 23.2 independent data points. The fit has 13.2 degrees of freedom.

	N (Nominal)	σ^2 ($Å^2$)	R (Å)
Pu–O	8	0.0025(9)	2.491(7)
Pu–C/N	8	0.02(1)	3.4(5)
Pu–C	10	0.02(3)	4.6(3)
MS (combined)	30	0.05(5)	3.7(2)
ΔE_0		-20.1(1) eV	
S_0^2		0.96(20)	
R (%)		14.7	

Table S3 **EXAFS fit results from Pu^{III}–343–HOPO**. Fit range is between 1.5 and 5.0 Å. The k^3-weighted data are transformed between 2.5-12.0 $Å^{-1}$ and are Gaussian narrowed by 0.3 $Å^{-1}$. The data have 23.2 independent data points. The fit has 13.2 degrees of freedom. Also shown are DFT results for the ground state Pu^{IV}-343-HOPO complex from Kelley *et al*. (Kelley *et al.*, 2018). Where the Debye-Waller factor σ^2 is reported for the EXAFS data, which includes both static and thermal contributions to the bond length distribution width, "var" represents the variance in the bond length distribution for the given shell from the DFT calculation, and includes no thermal contribution. Note that the values of *N*, apart from the Pu-N-O path, are fixed to their nominal values. This constraint was necessary since strong correlations between the *N*- and the σ^2-values for, primarily, the Pu-C/N and the Pu-N-O shells resulted in very large estimated errors. * = values constrained to avoid correlations between the Pu-C/N shell and the MS path.

	DFT			EXAFS		
	N	var ($Å^2$)	R (Å)	N	σ^2 ($Å^2$)	R (Å)
Pu–O	8	0.007	2.456	8	0.0037(6)	2.498(5)
Pu–C/N	8	0.0013	3.314	8*	0.01136*	3.348*
Pu–C	10	0.0067	4.682	10	0.010(4)	4.50(3)
Pu-N-O	30	0.029	3.682	25(5)	0.00250	3.54(2)
					ΔE_0 = -20.9(9) eV<br>S_0^2 = 1.02(7)<br>R(%) = 8.5	

S3. References

Ankudinov, A. L., Ravel, B., Rehr, J. J. & Conradson, S. D. (1998). *Phys. Rev. B* **58**, 7565-7576.

Booth, C. H. & Bridges, F. (2021). *International Tables for Crystallography*, Vol. I, doi:10.1107/S1574870720003444.

Booth, C. H. & Hu, Y.-J. (2009). *J. Phys.: Conf. Ser.* **190**, 012028.

Kelley, M. P., Deblonde, G. J. P., Su, J., Booth, C. H., Abergel, R. J., Batista, E. R. & Yang, P. (2018). *Inorg. Chem.* **57**, 5352-5363.

Li, G. G., Bridges, F. & Booth, C. H. (1995). *Phys. Rev. B* **52**, 6332-6348.
